# The effects of continuous catheter adductor canal block for pain management in knee replacement therapy: a meta-analysis

**DOI:** 10.1186/s43019-023-00188-0

**Published:** 2023-06-01

**Authors:** Aiden Jabur, Hyun Jae Nam, Asher Dixon, Tynan Cox, Hayden Randall, Jing Sun

**Affiliations:** 1 School of Medicine and Dentistry, Griffith University, Gold Coast, Australia; 2 Institute for Integrated Intelligence and Systems, Griffith University, Gold Coast, Australia; 3 School of Medicine, University of Sydney, Sydney, Australia

**Keywords:** Total knee arthroplasty, Single shot adductor canal block, Continuous technique adductor canal block, Pain management, Meta-analysis

## Abstract

**Purpose:**

Adductor canal block has emerged as a favourable element of multimodal analgesia regimens for total knee arthroplasty, due to the exclusive sensory blockade it provides. However, it is controversial as to whether a single shot or continuous technique adductor canal block is superior. This meta-analysis examined the effect of both these techniques on pain management associated with total knee arthroplasty.

**Methods:**

All randomised controlled trials published on Cochrane Library, PubMed, and EMBASE, Scopus, and PsychINFO were systematically searched. The PEDro scale was used to assess the quality of studies. A total of 8 articles, 2 of which were split by subgroup analyses to create 10 studies, with 828 adults were selected for inclusion in the analysis. The mean difference and effect size with a 95% confidence interval (CI) were analysed for the pooled results.

**Results:**

Statistically significant pooled effects of analgesia technique in favour of catheter use were found in the reduction of pain scores and VAS scores, and total rescue analgesia dosage. No significant changes were observed in the hospital stay time. Subgroup analysis revealed that patients with BMI 30 or more reported higher pain scores than those with BMI below 30.

**Conclusion:**

Based upon studies that are currently available, our meta-analysis appears to demonstrate that continuous administration of analgesia through an adductor canal catheter provides greater pain reduction in total knee arthroplasty than single shot analgesia. Despite these current findings, future studies with larger sample sizes and greater control of study parameters are required to confirm the current findings.

**Supplementary Information:**

The online version contains supplementary material available at 10.1186/s43019-023-00188-0.

## Background

Post-operative pain control after total knee arthroplasty (TKA) remains a significant issue, as the procedure is one of the most frequently performed orthopaedic operations and can cause intense early postoperative pain leading to patient dissatisfaction [1–5].

Multiple analgesic regimens are described in the literature for TKA, consisting of preoperative, intraoperative and postoperative options. In current practice, a combination of these options is used, typically in a multimodal fashion involving oral analgesics, regional nerve block, local infiltration, and patient-controlled analgesia depending on surgeon preference. Among intraoperatively-administered pain management options, the femoral nerve block (FNB) is regarded as the gold standard by some for its ample reduction in pain, reduction in opioid use and shortened hospital length of stay [6]. However its associated quadriceps muscle weakness resulting from blockade of motor efferents to anterior thigh muscles hinders early mobilisation and rehabilitation [7, 8]. Local infiltration analgesia involves periarticular and intraarticular injection of a local anaesthetic cocktail. While there is a lack of consensus on the medications used and technique, it has demonstrated superior postoperative pain reduction [9] and reduced muscle weakness compared to FNB and is hence used widely for TKA [10]. The adductor canal block (ACB) has gained traction for this indication as a pure sensory block, targeting only the saphenous nerve and part of the obturator nerve [11]. Furthermore, the ACB has been previously shown to improve post-operative ambulation and quadriceps strength [12]. The two main types of ACB technique are the single shot ACB (SACB) using a single bolus of analgesic, and the continuous ACB (CACB) using continuous infusion of repeated boluses at specific intervals via catheter [13, 14]. However, there is currently no consensus as to which of these techniques provides superior pain relief and subsequent return to mobility [11]. Given the benefits of early mobilisation on long-term pain, range of motion, and risk of deep vein thrombosis, there is great utility in optimising one’s ambulation following TKA [7].

Whilst previous research has demonstrated a relative benefit of CACB over single shot ACB [15], there exists contradictory evidence suggesting that there is minimal or no benefit of the continuous injection method over single shot [16–18] and hence, it is currently unclear which subgroup of patients derive the most benefit from continuous therapy. Therefore, this paper aims to fill in a research gap by not only assessing the efficacy of CACB compared to SACB for pain management using comparisons between pain scores, but also including a larger number of studies and to include subgroup analysis to identify the sources of the efficacy of the CACB over SACB. There will be a focus on patient characteristics including age and body-mass index (BMI) using subgroup analyses. It is anticipated that CACB will be more effective in reducing postoperative pain than SACB, leading to decreased rescue analgesia usage and hospital stay time, which will aid in optimising patient outcomes after TKA.

## Materials and methods

### Search methods

The meta-analysis protocol was registered with the Prospero International Prospective Register of Systematic Reviews (Registration number CRD42020200119). The systematic literature review and meta-analyses were performed and reported according to the Preferred Reporting Items for Systematic Reviews and Meta-Analyses (PRIMSA) guidelines. We performed a literature search to identify published RCTs investigating single short versus continuous technique adductor canal block for postoperative analgesia in total knee arthroplasty surgery. Search strategies were designed in accordance with the PICO (Patients, Intervention, Comparator, Outcome) algorithm [19].

The population was adults aged 18 years or older with total knee replacement therapy or total knee arthroplasty. The intervention was single shot analgesia infusion within the intervention group. The control included patients who received continuous analgesia infusion. The primary outcomes considered pain measured by Visual Analogue Score (VAS) and Numeric Rating Scale (NRS), with secondary outcomes including total rescue analgesia dosage and hospital stay time.

The keywords used in the search for relevant studies were as follows: Single AND (Continuous OR Catheter) AND (Adductor Canal Block OR Adductor Canal Blockade) AND (Total Knee Replacement OR Knee Arthroplasty) AND Randomised Control Trial, using employed medical subject headings (MeSH). No restrictions or filters were used.

### Inclusion criteria

Inclusion of studies within the meta-analysis followed strict criteria as follows: (1) Published in a peer-reviewed journal in the past 10 years (August 2010 until August 2020); (2) RCT study design; (3) participants were adult patients aged 18 years or older with total knee replacement therapy or total knee arthroplasty; (4) single shot analgesia or continuous infusion analgesia were used for anaesthetic induction; (5) primary outcome variables included VAS and NRS, with total rescue analgesia dosage and hospital stay time as secondary outcomes; (6) if multiple studies were published on the same population only the most recent study was included.

### Data extraction

**Fig. 1:**
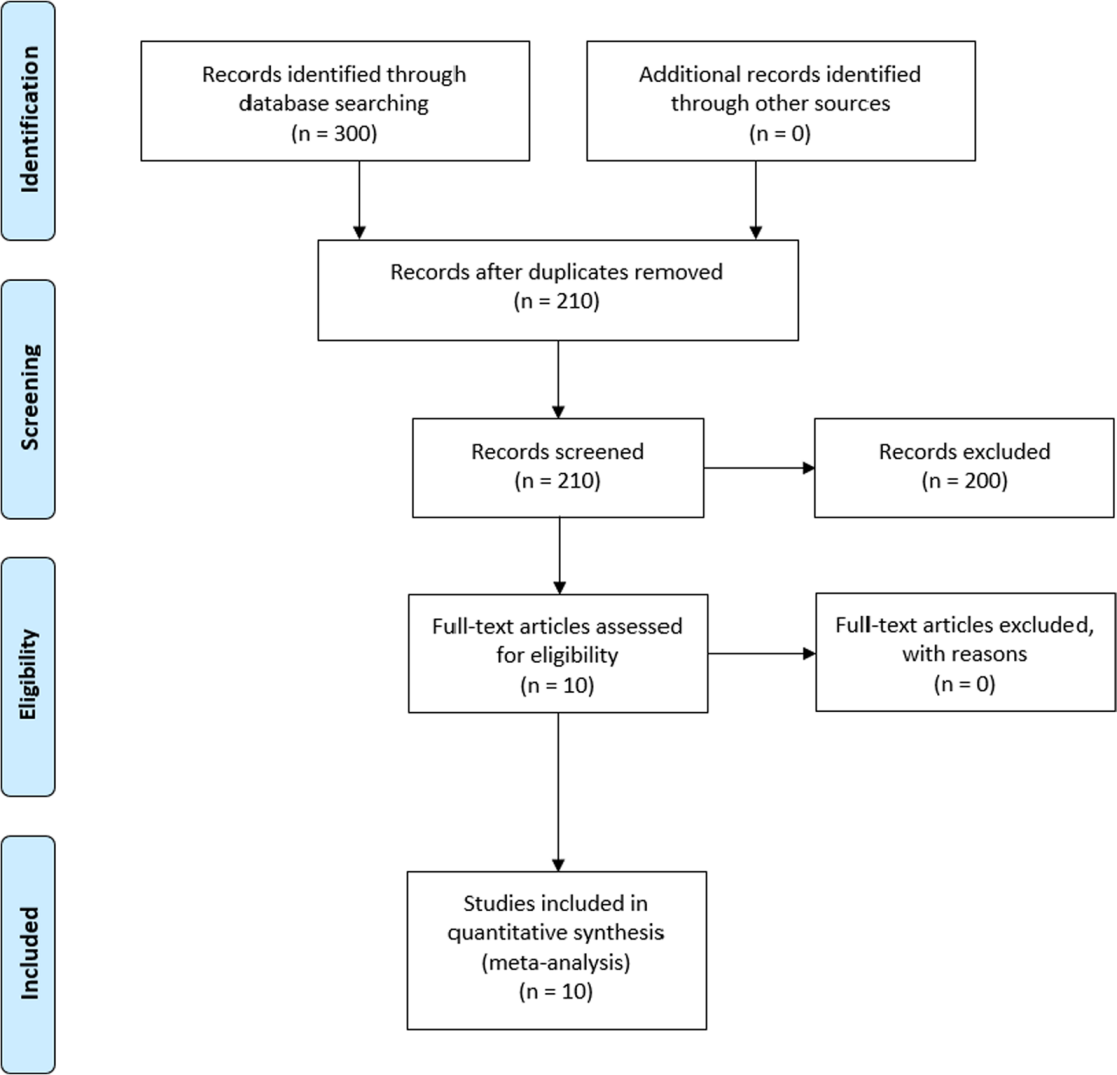
PRISMA flowchart of included studies

Two authors completed the initial search with review of each search strategy, with TC conducting the first search and HM conducting a parallel search. AJ was responsible for resolving any disagreements in the discussion in the search. All citations and abstracts where possible were downloaded to EndNote X9 for review. The databases searched were Cochrane Library, PubMed, and EMBASE, Scopus, PsychINFO. Scientific articles in English and Chinese reporting original data of RCTs published in peer-reviewed journals were evaluated. Studies were excluded where study design and methodology were unclear or did not sufficiently describe the intervention, or if a non-standardised delivery protocol was used. Duplicates and articles for which the full text was not available were excluded. Studies that included multiple sub-studies were considered in the search as separate individual studies. All search results underwent a primary screening process, performed independently by two reviewers, based on title and abstract according to the inclusion criteria (Fig. 1). Among the 10 studies included in the meta-analysis, there were two studies that contained two sub-studies. Data with regard to study design; study location; number of participants; participant age; participant sex; participant BMI; adductor canal block method (single-injection or continuous-injection) and total duration of infusion; type of rescue analgesia used; patient reported pain scores using VAS or NRS collected as the mean and standard deviation at 2, 4, 8, 12, 24, 48, 72 h for NRS and 4, 8, 12, 24, 48, and 72 h for VAS; total rescue analgesia dosage were extracted from the 10 studies. Furthermore, all VAS and NRS scores were converted to a rating from 0 to 10 to ensure comparability between studies that use different pain scores [20, 21].

Data regarding study setting, design location, study blinding, study population, sample size, interventions, comparators, potential biases in the conduct of the trial, and outcomes were extracted from eligible publications.

### Quality assessment

The Physiotherapy Evidence Database tool (PEDro scale) was used to assess the external validity (criteria 1), internal validity and biases (criteria 2–9), and the interpretability of the findings (criteria 10–11) of the randomised control trials. The criteria are as follows: specified eligility criteria, random allocation, allocation concealment, blinding of subjects, blinding of clinicians, blinding of researchers, key outcome measurement in 85% of initial subjects, intention to treat, between group comparison, measure of variability, and similarity at baseline. The PEDro tool aims to categorise studies into three quality levels: low quality (≤ 3 points), moderate quality (4–7 points), high quality (≥ 8 points). Eligible papers were all analysed independently by two reviewers, HN and KR, and any discrepancies were resolved via discussion (Additional file 1: Table S1).

### Statistical analysis

Statistical analysis was performed using Comprehensive Meta-Analysis Version 4 (Biostat, Englewood, NJ 2022). Subgroup analyses were performed using age (< 70 years compared with ≥ 70 years) and BMI (< 30 BMI for normal and overweight patients compared with ≥ 30 BMI for obese patients) based on WHO definitions. The age cut-off of 70 years was chosen since patients using catheter above this age are at high risk of developing negative health outcomes [22]. The effect of continuous catheter compared to single shot adductor canal blocks was described with the use of a pooled effect size method. Random effect meta-analyses were performed to describe the overall effect size. The mean difference was used to present the effect size for each intervention and time frame. Analysis of each study to assess for inconsistency included visual assessment of confidence intervals for overlap and *I*^2^ statistics of heterogeneity.

## Results

In the initial search, 300 articles were identified from the key databases (Cochrane Library, PubMed, EMBASE, and Scopus, PsychINFO) (Fig. 1). All articles were imported into Endnote, and 90 duplicate articles were removed. The remaining 210 studies were screened by review of title and abstract, removing 200 articles according to the inclusion and exclusion criteria. The remaining 10 studies were included in the final quantitative analysis; 4 were of high quality (8 points or more) and 6 were of moderate quality (5–7 points) by PEDro analysis. Table 1 shows the final list of included studies and their summary characteristics.

**Table 1:** Characteristics of included studies

Studies	Participants (*n*) I/C	Design; Location	Age (mean, SD) I/C	Sex (F, M) I/C	BMI (mean, SD) (kg/m^2^), I/C	Pre-emptive analgesia	Anaesthesia type and tourniquet status	SACB method and dose	CACB method and dose	Rescue analgesia
Canbek et al. 2019	63 / 60	SB RCT; Turkey	66.9, 6.8 / 67.1, 6.9	15, 48 / 10, 50	31.4, 4.8 / 32.3, 4.3	Diclofenac sodium 75 mg or Paracetamol 1 g (if serum creatinine abnormal)	Spinal with tourniquet	Immediately postop bupivicaine 0.25% 30 mL	Immediately postop bupivacaine 0.125% 5 mL/h (total 125 mL over 24 h)	Tramadol (50 mg)
Elkassabany et al. 2019	53 / 51	NB RCT; USA	63.9, 9.6 / 66.5, 8.5	37, 16 / 29, 22	31.5, 5.1 / 31.2, 5.2	-	Spinal: SACB *n* = 40 CACB *n* = 39 With tourniquet	Intraoperative ropivacaine 0.5% 20 mL	Intraoperative ropivacaine 0.5% 17–18 mL bolus + 0.2% 8 mL/hr (24 h)	Oxycodone IV
Elkassabany et al. 2019	53 / 52	NB RCT; USA	63.9, 9.6 / 62.2, 8.7	37, 16 / 34, 18	31.5, 5.1 / 31.9, 4.9	-	Spinal: SACB *n* = 40 CACB *n* = 43 With tourniquet	Intraoperative ropivacaine 0.5% 20 mL	Intraoperative ropivacaine 0.5% 17–18 mL bolus + 0.2% 8 mL/hr (48 h)	Oxycodone IV
Kim et al. 2019	22 / 22	NB RCT; South Korea	66.4, 8.8 / 70.1, 10.3	2, 20 / 3, 19	27.1, 4.1 / 25.5, 3.9	None given	General with tourniquet	1 h preoperative ropivacaine 0.5% 20 mL bolus + IV fentanyl 0.4 µg/kg/h	1 h preoperative ropivacaine 0.5% 5 mL bolus + 0.2% 5 mL/hr	Tramadol (50 mg)
Lyngeraa et al. 2019	49 / 49	DB RCT; Denmark	69.7, 8.5 / 70.3, 8.8	13, 37 / 21, 30	28.7, 4.7 / 28.4, 4.9	Paracetamol 1 g Celecoxib 400 mg	Spinal with tourniquet	Immediately postop ropivacaine 0.75% 20 mL	Immediately postop ropivicaine 0.75% 20 mL bolus + standard catheter 0.2% 20 mL every 8 h until 12 pm POD2	Morphine IV
Lyngeraa et al. 2019	49 / 52	DB RCT; Denmark	69.7, 8.5 / 70.4, 6.9	13, 37 / 21, 31	28.7, 4.7 / 28.3, 4.7	Paracetamol 1 g Celecoxib 400 mg	Spinal with tourniquet	Ropivacaine 0.75% 20 mL	Ropivicaine 0.75% 20 mL bolus + suture-method catheter 0.2% 20 mL every 8 h until 12 pm POD2	Morphine IV
Li et al. 2017	30 / 30	NB RCT; China	67.7, 7.8 / 65.9, 8.4	6, 24 / 6, 24	24.2, 2.7 / 25.2, 3.2	Celecoxib 200 mg twice daily for 3 days	General With tourniquet	Ropivacaine 2.5 g/L 30 mL + Adrenaline 0.1 mg	Ropivacaine 2.5 g/L 8 mL/hr + 5 mL (After 48 h and stays for 30 min)	Pethidine hydrochloride (50 mg)
Shah et al. 2015	39 / 46	DB RCT; India	66.3, 6.38 / 68.34, 7.71	7, 32 / 13, 33	30.27, 5.4 / 29.58, 5.55	Diclofenac sodium 75 mg 8 hourly or Paracetamol 1 g 8 hourly (if serum creatinine abnormal)	Spinal No tourniquet	Ropivacaine 0.75% 30 mL + saline 30 mL 4 h post-op	Ropivacaine 0.75% 30 mL bolus + 0.25% 30 mL every 4 h until 8am POD2	Tramadol (50 mg) IV
Turner et al. 2018	30 / 30	DB RCT; USA	68.8, 10 / 70.9, 7.9	21, 9 / 13, 17	31.3, 5 / 31.5, 6	Paracetamol 1 g Pregabalin 150 mg Celecoxib 400 mg	Spinal or general Tourniquet status not reported	Bupivacaine 0.25% + clonidine 1.67 µg/mL + dexamethasone 2 mg + buprenorphine 150 µg + epinephrine 2 µg/mL, for a total 20 mL bolus	Bupivacaine 0.25% + epinephrine 2.5mcg/mL for a total 20 mL bolus + bupivacaine 0.125% 8 mL/h continued through to POD2	Oxycodone, Hydromorphone IV
Zhang et al. 2018	25 / 23	DB RCT; China	65, 8 / 67, 7	4, 21 / 5, 18	25.96, 3.38 / 26.32, 4.25	–	–	Ropivicaine 0.5% 20 mL + intermittent saline 12 and 24 h post-operative	Ropivicaine (standard catheter, 0.5%) 20 mL pre-operative + 0.5% 20 mL at 12 h and 24 h post-operative	Pethidine hydrochloride (50 mg) IM

*I/C*  Intervention Group / Control Group, *SACB*  Single-injection adductor canal block, *CACB*  continuous-injection adductor canal block, *VAS*  Visual Analog Scale, *NRS*  Numeric Rating Scale, *POD*  post-operative day, *NB* non-blinded, *SB* single blinded, *DB*  double blinded

### Participants

Table 1 provides an outline of included studies. Outcomes were reported on 828 adults receiving single injection (*n* = 413) or continuous technique (*n* = 415) adductor canal block. Method of adductor canal block was used to assess different postoperative outcomes, with eight studies measuring pain as VAS score [13, 17, 18, 23–25]; two studies measuring pain as NRS score [26, 27]; five studies measuring total rescue analgesia dosage [13, 18, 26, 27]; four studies measuring hospital stay time [13, 18, 27]; six studies measuring physical therapy endpoints [18, 23, 26, 27]; three studies measuring quality of recovery [18, 26]; two studies measuring adverse effects [26, 27]; and one study measuring patient satisfaction [27]. These studies were conducted in 6 different locations, including Turkey [13], USA [18], South Korea [26], Denmark [28], China [18, 23], and India [17]. Duration of infusion in continuous technique adductor canal block groups varied, with three studies reporting 24 h [13, 18, 23], six studies reporting 48 h [17, 18, 25, 27, 29], and one study did not report the duration [26]. No data was available from the studies to directly compare the study population’s baseline demographics or surgical techniques.

### Effect on outcome variables

As shown in Table 2, there were significant differences between single shot and continuous technique adductor canal block in pain scores in 2 h, 4 h, 8 h, 12 h, 24 h, 48 h and 72 h in both standardised mean difference (effect size) and mean difference. There were also significant differences between single shot and continuous technique adductor canal block in pain scores measured using the VAS scale in 4 h, 8 h, 12 h, 24 h, 48 h and 72 h in both standardised mean difference (effect size) and mean difference. There were significant differences in both standardised mean difference (0.275; 95% CI 0.068, 0.483; *p* < 0.01) (effect size) and mean difference (11.237; 95% CI 3.574, 18.899; *p* < 0.01) between single shot and continuous technique adductor canal block in total rescue analgesia, and no statistically significant difference in hospital stay time between the two groups (mean difference 0.071; 95% CI − 0.031, 0.174; *p* > 0.05).

**Table 2:** Result of all variable analysis of included studies in meta-analysis

Variables	Studies (*n*)	Participant (*n*)	Mean difference			Effect size			Publication bias
			MD (95% CI)	*Q* test	*I*^2^(%)	Effect size (95% CI)	*Q* test	*I*^2^(%)	Egger’s *t* value (95% CI)
2-h pain score	3	227	0.966 (0.057, 1.876) *	21.408	90.658 ***	0.791 (0.480, 1.103) ***	2.503	20.104	0.238 (− 53.858, 51.879)
4-h pain score	5	360	1.153 (0.480, 1.826) ***	93.987	95.744 ***	1.522 (0.552, 2.491) **	63.631	93.714 ***	1.324 (− 10.004, 24.254)
8-h pain score	6	511	0.839 (0.381, 1.297) ***	49.079	89.812 ***	1.084 (0.364, 1.803) **	70.278	92.885 ***	2.126 (− 3.005, 22.656)
12-h pain score	4	328	0.999 (0.282, 1.716) **	12.994	76.912 **	0.720 (0.249, 1.190) **	12.497	75.994 **	0.875 (− 40,558, 26.853)
24-h pain score	8	619	0.884 (0.274, 1.494) **	87.891	92.036 ***	0.892 (0.441, 1.344) ***	42.381	85.843 ***	2.347 (− 0.362, 17.285)
48-h pain score	7	534	0.892 (0.441, 1.344) ***	42.381	85.843 ***	1.005 (0.432, 1.578) ***	56.123	89.309 ***	2.474 (− 0.292, 15.262)
72-h pain score	3	231	0.556 (0.131, 0.981) *	3.738	46.501	0.511 (0.181, 0.840) **	2.930	31.737	1.185 (− 44.972, 37.301)
4-h VAS	4	316	1.956 (0.232, 3.680) *	43.757	93.144 ***	0.792 (0.229, 1.356) **	0.520	82.149 ***	3.190 (− 33.256, 4,939)
8-h VAS	5	467	2.822 (0.555, 5.090) *	46.274	91.356 ***	0.648 (0.110, 1.186) *	31.805	87.423 ***	0.340 (− 35.898, 44.461)
12-h VAS	3	268	2.934 (0.738, 5.130) **	25.189	92.060 ***	0.929 (0.581, 1.277) ***	3.637	45.007	0.291 (− 99.140, 94.701)
24-h VAS	6	515	1.965 (0.634, 3.297) **	31.177	83.963 ***	0.561 (0.165, 0.956) **	23.922	79.099 ***	0.555 (− 15.519, 20.280)
48-h VAS	5	430	0.996 (0.136, 1.856) *	17.271	76.840 **	0.553 (0.344, 0.763) ***	4.633	13.664	1.540 (− 12.257, 4.262)
72-h VAS	3	231	0.556 (0.132, 0.981) *	3.735	46.457	0.511 (0.181, 0.840) **	2.930	31.751	1.185 (− 44.977, 37.305)
Total rescue analgesia	5	361	11.237 (3.574, 18.899) **	1.860	0	0.275 (0.068, 0.483) **	2.908	0	1.007 (− 4.340, 8.354)
Hospital stay time	3	268	0.071 (− 0.031, 0.174)	1.043	0	0.131 (− 0.109, 0.371)	1.734	0	0.927 (− 14.890, 12.866)

*P* < 0.05, *; *P* < 0.01, **; *P* < 0.001, ***

Adverse effects of treatments such as nausea and vomiting was monitored by 8 out of 10 studies [13, 17, 18, 23, 25, 26]. Lyngeraa et al. [24] and Lyngeraa et al. [24] [29] did not monitor any adverse effects of treatments. Canbek et al. and Kim et al. reported that there were no adverse effects of treatments in both SACB and CACB groups [13, 26]. Elkassabany et al. (2019) reported adverse effects as a sum of scores as a part of the Revised American Pain Society Patient Outcome Questionnaire (APS-POQ-R), where the SACB group scored 9 (3 to 16), 24 h CACB group scored 11 (5 to 16), and 48 h CACB group scored (7 to 15) [18]. Li et al. reported 6 adverse effects in the SACB group (3 nausea, 2 vomiting, and 1 drowsiness), and 3 adverse effects in the CACB group (1 nausea, 2 vomiting) [25]. Shah et al. reported 1 adverse effect in both the SACB and CACB groups [17]. Turner et al. reported adverse effects by postoperative days (POD), where there were 4 adverse effects on postoperative day 1 and no increase in adverse effects on POD2 in the SACB group, and 7 adverse effects on postoperative day 1 and 10 total adverse effects by POD2 in the CACB group [27]. Zhang et al. reported 3 adverse effects in the SACB group, and 4 adverse effects in the CACB group [23].

Subgroup analysis found only age group explained the significant difference between single shot and catheter group in the pain score in 8-h, 24 h when people were aged less than 70 years had more pain score than people who aged 70 or more. However, when pain score was measured at 48 h, people who were aged 70 or more had more pain score than people who aged less than 70. Additionally, people with a BMI of 30 or more had higher pain scores than people with a BMI lower than 30 when pain scores were measured at both 24 h and 48 h.

**Fig. 2:**
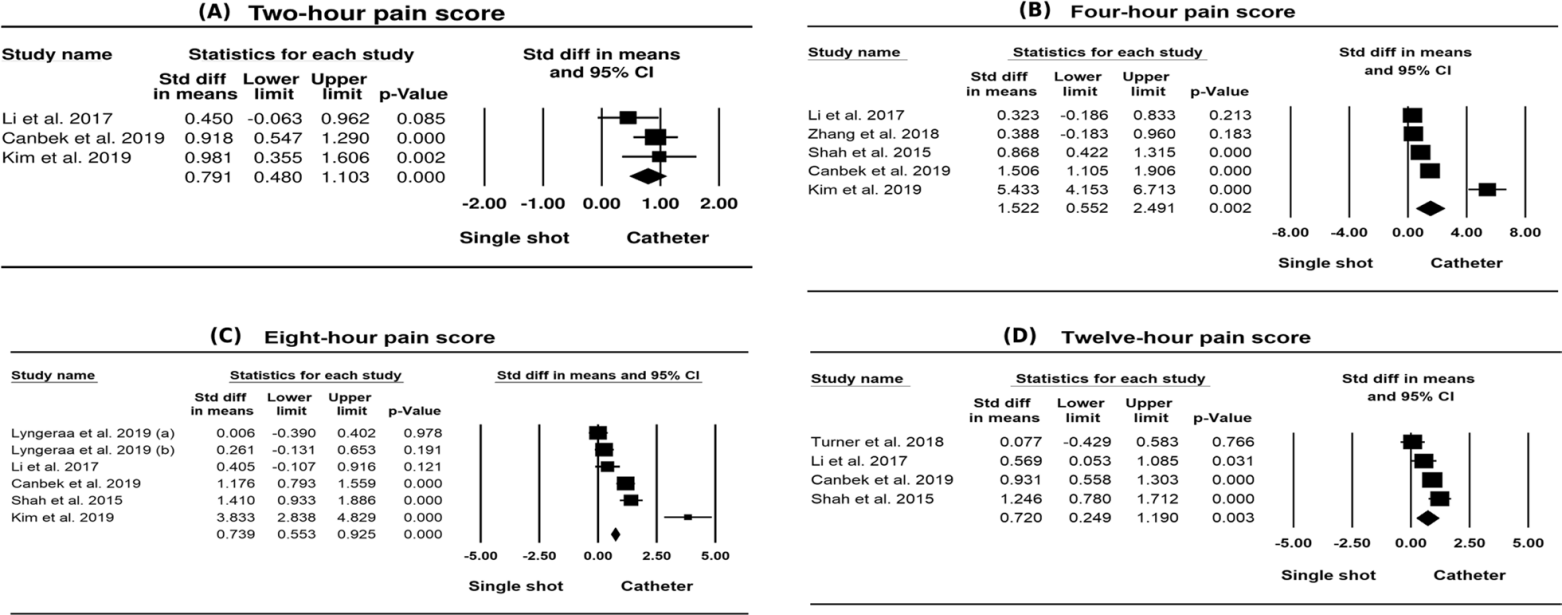
Forest plots in 2 h, 4 h, 8 h, and 12 h pain score

**Fig. 3:**
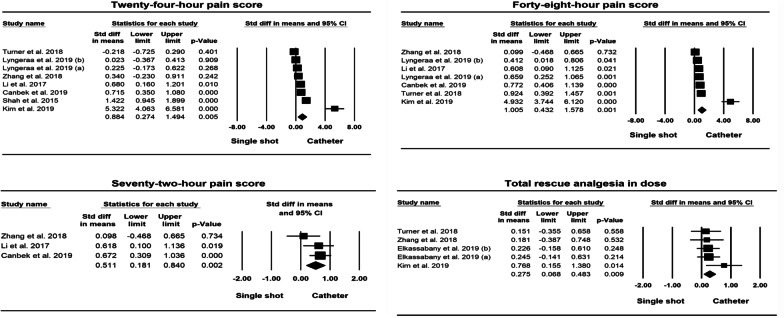
Forest plots in 24 h, 48 h and 72 h pain score, and total rescue analgesia dosage in milligrams (mg)

**Fig. 4:**
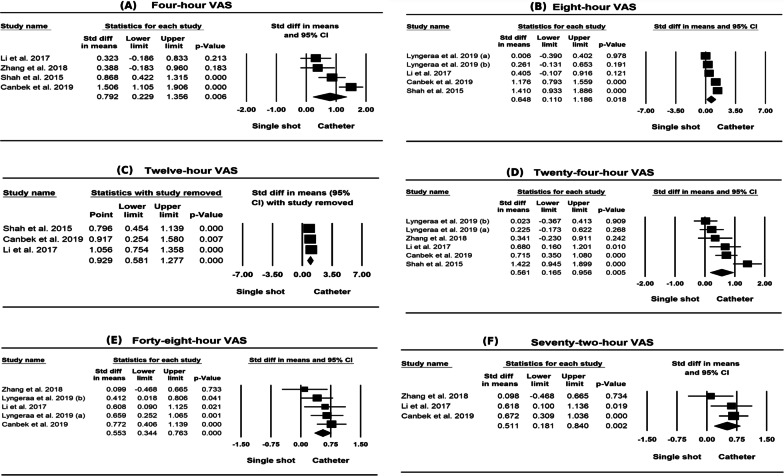
Forest plots in 4 h, 8 h, 12 h, 24 h, 48 h, and 72 h VAS score

Egger regression analysis showed that all pain scores and VAS scores had *P* value more than 0.05 suggesting there was no publication bias (Figs. 2, 3, 4).

 Sensitivity analysis demonstrated that the overall results remained significant when removing a study per time suggesting the results were not due to any single study (Tables 3, 4).

**Table 3:** Subgroup analysis for included studies

Subgroups	Studies (*n*)	Participant (*n*)	Mean difference			Effect size		
			Mean difference (95% CI)	*Q* test	*I*^2^(%)	Effect size (95% CI)	*Q* test	*I*^2^(%)
Eight-hour pain score age group								
< 70	3	268	1.238 (0.175, 2.301) *	25.271	92.086 ***	1.009 (0.461, 1.558) ***	8.729	77.089 **
70 and more	3	243	0.546 (-0.210, 1.302)	18.279	89.059 ***	1.266 (-0.209, 2.741)	49.998	96.000 ***
Twenty-four-hour pain score age group								
< 70	4	316	0.809 (0.445, 1.173) ***	5.252	42.883	0.802 (0.380, 1.224) ***	9.427	68.177 *
70 and more	4	303	0.402 (-0.523, 1.326)	35.166	91.469 ***	1.131 (-0.086, 2.347)	66.888	95.515 ***
Forty-eight-hour pain score age group								
< 70	3	231	0.635 (0.099, 1.172) *	5.446	63.278	0.538 (0.157, 0.919) **	3.836	47.856
70 and more	4	303	1.083 (0.473, 1.692) ***	24.334	87.672 ***	1.549 (0.465, 2.632) **	50.873	94.103 ***
Twenty-four-hour pain score BMI group								
< 30	6	467	0.690 (0.203, 1.177) ***	56.129	91.092 ***	1.156 (0.325, 1.986) ***	78.148	93.602 ***
30 and more	2	152	0.492 (-1.265, 2.248) **	7.945	87.414**	0.266 (-0.647, 1.179) **	8.554	88.310 **
Forty-eight-hour pain score BMI group								
< 30	5	382	0.760 (0.169, 1.351) ***	39.337	89.831 ***	1.158 (0.284, 2.033) *	54.959	92.722 ***
30 and more	2	152	1.251 (0.402, 2.100) ***	2.652	62.297	0.821 (0.519, 1.123) **	0.214	0.000

*CI* confidence interval

*P* < 0.05, *; *P* < 0.01, **; *P* < 0.001, ***

**Table 4:** Egger regression results for publication bias

Variable	Egger test	*P*
24 h pain score	3.449 (3.381, 19.892)	0.014
48 h pain score	3.215 (2.233, 20.055)	0.024
Total rescue analgesia dosage	1.007 (− 4.340, 8.354)	0.388
2 h pain score	5.982 (− 17.523, 48.698)	0.105
4 h pain score	2.498 (− 3.934, 32.673)	0.088
8 h pain score	2.659 (− 0.640, 29.647)	0.056
12 h pain score	1.594 (− 34.984, 76.178)	0.252
72 h pain score	1.772 (− 65.142, 86.259)	0.327
Hospital stay time	0.201 (− 82.330, 84.983)	0.873
VAS 4 h	1.005 (− 401.054, 469.960)	0.498
VAS 8 h	1.552 (− 23.773, 69.056)	0.218
VAS 24 h	2.589 (− 4.826, 46.946)	0.081
VAS 48 h	1.081 (− 44.118, 73.736)	0.393

## Discussion

The capacity to minimise postoperative pain in TKA is significantly relevant for surgeons and patients as it affects postoperative rehabilitation [18, 30, 31]. CACB demonstrated a higher efficacy for pain management over SACB, with VAS scores between 4 and 24 h postoperative reaching the acceptable minimal clinically important difference (MCID) after TKA of 1 to 2 points [32–34]. It is plausible a major limitation of peripheral nerve blocks such as the SACB is the short duration of action, between 12 to 24 h [35]. The increased administration of rescue analgesia in the SACB group compared the CACB group is likely due to the SACB wearing off after 24 h, leading to increased rescue analgesia use after this period [26]. Furthermore, CACB allows for the delivery of greater doses of anaesthetic for longer durations of time, resulting in higher efficacy of postoperative pain management for a longer period of time [36]. Additionally, lower pain scores after 2 h in the CACB group compared to the SACB group can be attributed to the use of spinal anesthesia in some study groups, which may have exaggerated the impact of ACB, ultimately resulting in a larger difference in pain scores between CACB and SACB groups in the several hours after surgery [17, 26].On the other hand, CACB has unique complications compared to SACB, including catheter obstruction, migration, leakage of local anaesthetic, accidental removal, and rarely infection [35]. Risks of ACB generally include vascular puncture and bleeding, nerve damage, and local anaesthetic toxicity [35].

The statistically insignificant decrease in hospital stay time in the CACB group compared with the SACB group corroborates with previous studies showing that pain control is an important factor in decreasing hospital stay time after TKA [35, 37, 38]. Additionally, poor quadriceps muscle strength induced by ACB or inadequate pain management may worsen with prolonged continuous infusion due to the motor branch of the vastus medialis muscle contained within the adductor canal being affected, especially with high volumes and repeated infusions of anaesthetic [39], delaying patient rehabilitation after surgery and adversely impacting physiotherapy. This may result in a longer hospital stay time.

Our study is the first to make use of subgroup analysis by incorporating RCTs inclusive of the target population age and BMI. At 8 and 24 h postoperatively, the CACB has a significant effect size in patients under 70 years old, however this is insignificant in those aged 70 and over. At 48 h, both age groups benefit from lower pain scores through the CACB, yet this effect is more pronounced in the older age group. This may be explained by age-related slowing of drug metabolism and clearance related to decline in hepatic and renal function [41, 42]. At 24 and 48 h, significant pain score effect sizes were detected in both BMI groups when comparing SACB and CACB, and the effect is more pronounced in the < 30 BMI group. One theory for this is that increased α-acid glycoprotein in obesity reduces the free fraction of anaesthetic and increases the dose requirement for nerve block [45].

Although meta-regression analysis may have reduced confounding effects and yielded valuable insight into effects of anaesthesia type, tourniquet use, ACB technique and pre-emptive medication, there is insufficient power. Anaesthetic type was typically left to anaesthetists to decide, which is subject to local protocols and policies—three studies used exclusively spinal anaesthetic [13, 17, 29], two used general [23, 26], and two were mixed [18, 27]. All but one study used a tourniquet for the operation [17] and one failed to report [27]. CACB technique was using standard catheter, however one study also featured a suture-method catheter [29]. Pre-emptive medications also differed depending on local policy and anaesthetist/surgeon preference and is outlined in Table 1.

Cost-effectiveness is an important factor to consider, with adductor canal catheters being $80 compared to the relatively in-expensive SACB [23, 26, 29, 40]. There is also greater expertise required for CACB administration [26]. Decreased hospital stay time with CACB may ameliorate this and facilitate more efficient patient turnover, however to our knowledge no formal investigation has been conducted.

Patient satisfaction is inconclusive; one study reports decreased satisfaction with CACB [23], another reports increased satisfaction [18], and another states there is no difference [27]. This is likely due to differences in measurement methods—APS-POQ-R [18], 5-point Likert scale [27], willingness to recommend same treatment [27], and dichotomous verbal evaluation [23].

Findings from this meta-analysis contradicts some prior published findings in the literature. One meta-analysis has been published on single shot and continuous technique ACB after TKA, which found no significant difference in pain at 24 h postoperative, morphine consumption, risk of complications or length of hospital stay between the two analgesic approaches [16]. Our meta-analysis synthesised more evidence by including a larger number of studies (10, as opposed to 4 in the aforementioned meta-analysis), larger sample size, and novel subgroup analysis into body-mass index (BMI) and age, and also includes studies using both NRS and VAS pain scales.

A limitation of this study is the exclusion of RCT studies evaluating unicompartmental knee arthroplasty (UKA), reducing the power and potential clinical significance of the results. Furthermore, disparities in spinal and general anaesthesia between the studies as well as tourniquet use may be confounding factors for pain score and hospital stay time outcomes. There is also heterogeneity in the agents used for ACB, which are demonstrated in Table 1. Whilst discharge criteria are an important determinant of hospital stay time, these criteria were not outlined in the included studies, representing a limitation in our scope of interpretation of hospital stay time comparison. While this study focused on differences in pain, rescue analgesia and hospital stay time, additional secondary outcomes can be analysed such as physiotherapy endpoints and adverse effects like postoperative nausea and vomiting. These were not analysed in this study due to insufficient available data. Additionally, there was an inadequate number of studies to analyse the difference with type of local anaesthetic or additive agents and outcome. Further RCT studies are required to clarify findings.

In conclusion, continuous, or catheter-administered, ACB features may significantly lower pain scores slightly above the MCID and may significantly lower total rescue analgesia than single shot ACB, which suggests that catheter use may be the better approach to be applied in clinical settings after total knee replacement. The strength of this conclusion can be improved with greater evidence from studies with a robust methodology.

## Supplementary Information

**Additional file 1.** Quality of assessment for included studies using PEDro scale.

## Data Availability

Data will be available upon request.
