# Supplementary material for: The effects of continuous catheter adductor canal block for pain management in knee replacement therapy: a meta-analysis
Source: Knee Surg Relat Res. 2023 Jun 1;35:16. doi: 10.1186/s43019-023-00188-0 (PMC10236883; doi:10.1186/s43019-023-00188-0)

**Appendix**

Table S1. PEDro analysis of remaining Studies

| **Trial** | **Specified Eligibility Criteria** | **Random Allocation** | **Allocation Concealment** | **Blinding of Subjects** | **Blinding of Assessors** | **Blinding of Researchers** | **Similar at baseline** | **Key outcome measurement 85%** | **Intention to treat** | **Between group comparison** | **Measure of variability** | **Total score** |
| --- | --- | --- | --- | --- | --- | --- | --- | --- | --- | --- | --- | --- |
| Canbek et al. (2019) | X | X | - | - | X | - | X | X | X | X | X | 8/11 |
| Elkassabany et al. (2019) | X | X | - | - | - | - | X | X | X | X | X | 7/11 |
| Kim et al. (2019) | X | X | - | - | - | - | X | X | X | X | X | 7/11 |
| Li et al. (2017) | X | X | - | - | - | - | X | X | X | X | X | 7/11 |
| Lyngeraa et al. (2019) | X | X | - | - | - | - | X | X | X | X | X | 7/11 |
| Shah et al. (2015) | X | X | - | X | - | - | X | X | X | X | X | 8/11 |
| Turner et al. (2018) | X | X | - | X | - | X | X | X | X | X | X | 9/11 |
| Zhang et al. (2018) | X | X | - | X | - | X | X | X | X | X | X | 9/11 |
| Elkassabany et al. (2019) | X | X | - | - | - | - | X | X | X | X | X | 7/11 |
| Lyngeraa et al. (2019) | X | X | - | - | - | - | X | X | X | X | X | 7/11 |

X = characteristic within study, - = characteristic absent from study
Figure S1.
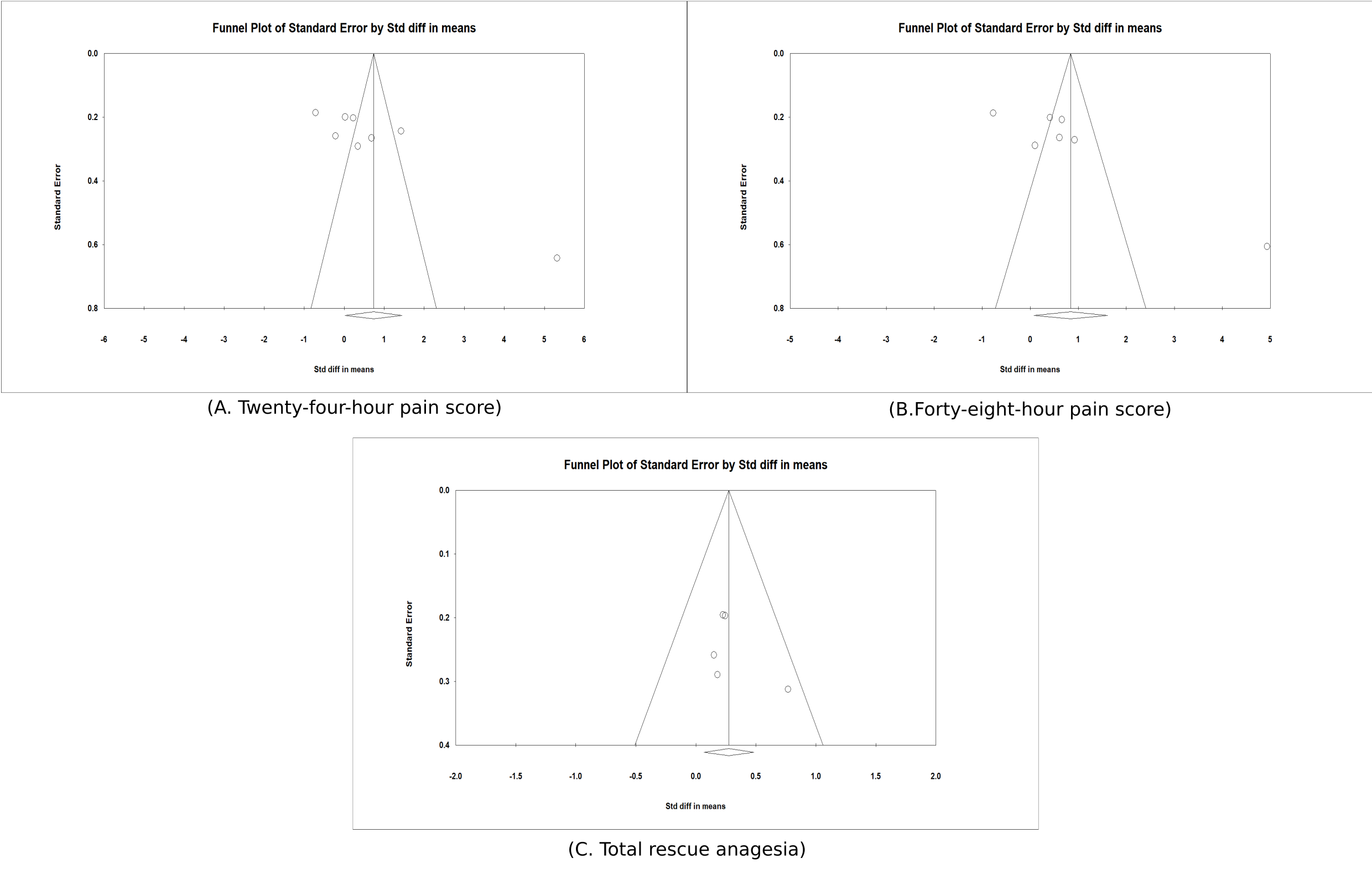


Figure S2.
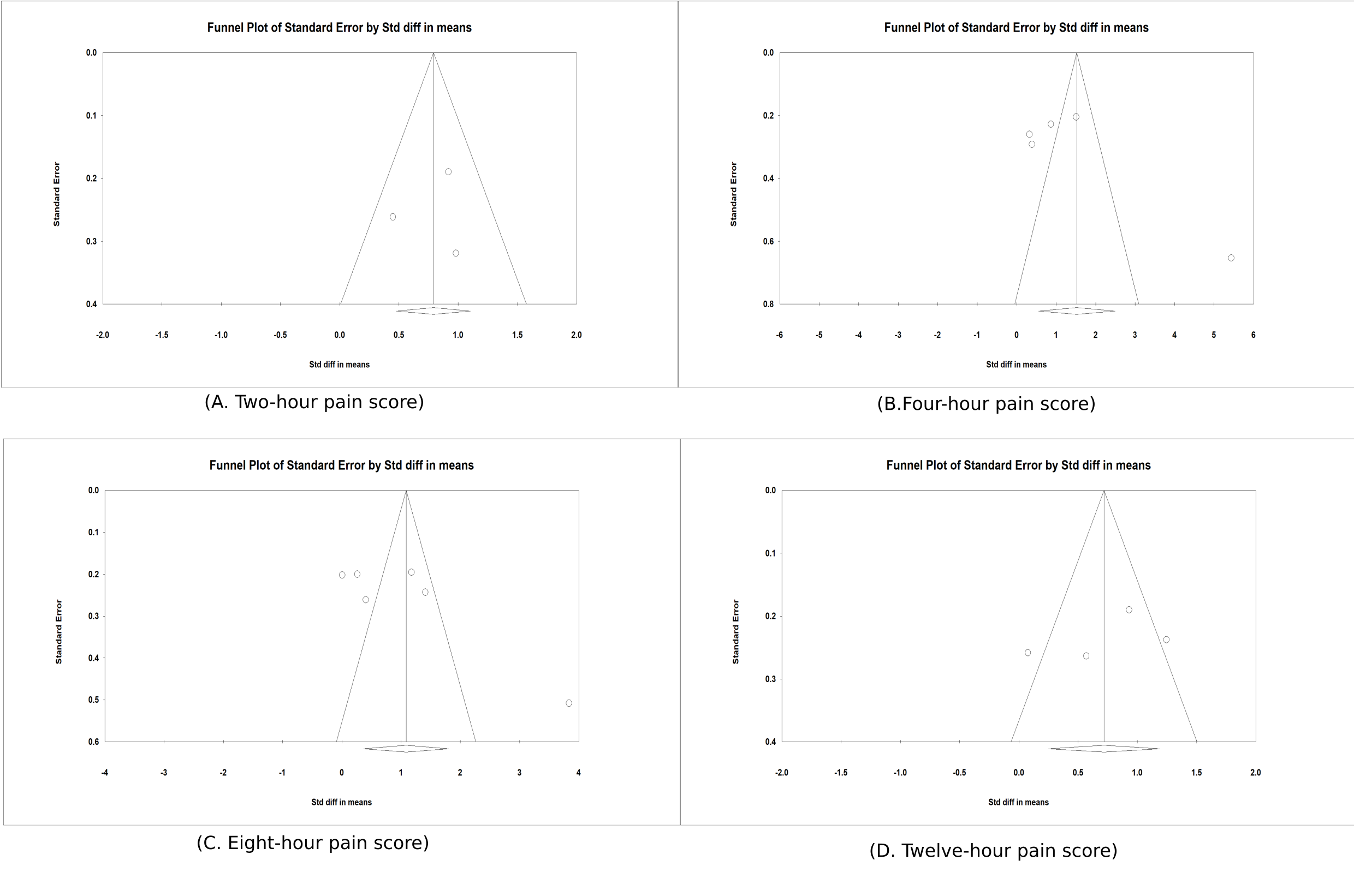


Figure S3.
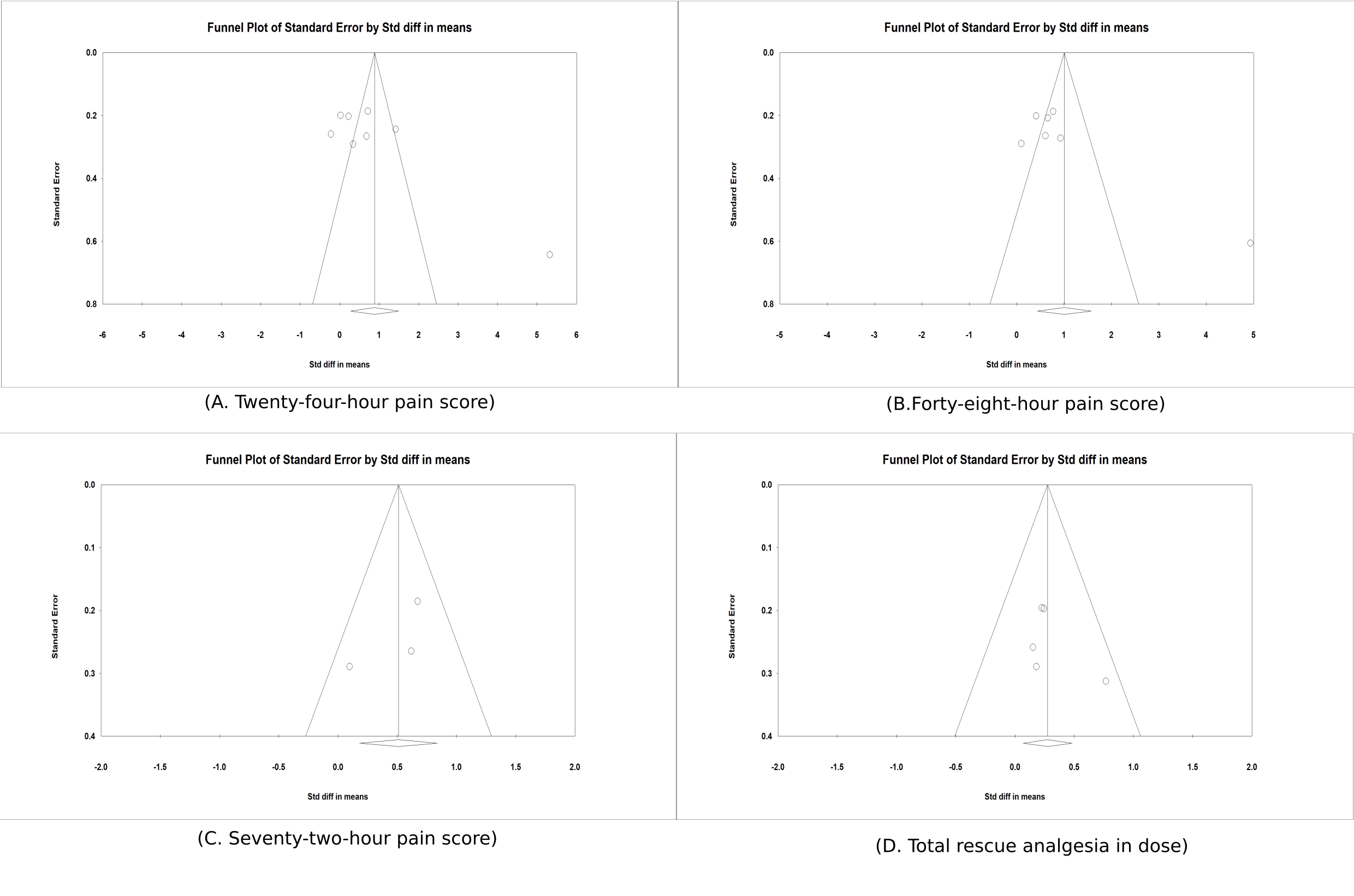


Figure S4.


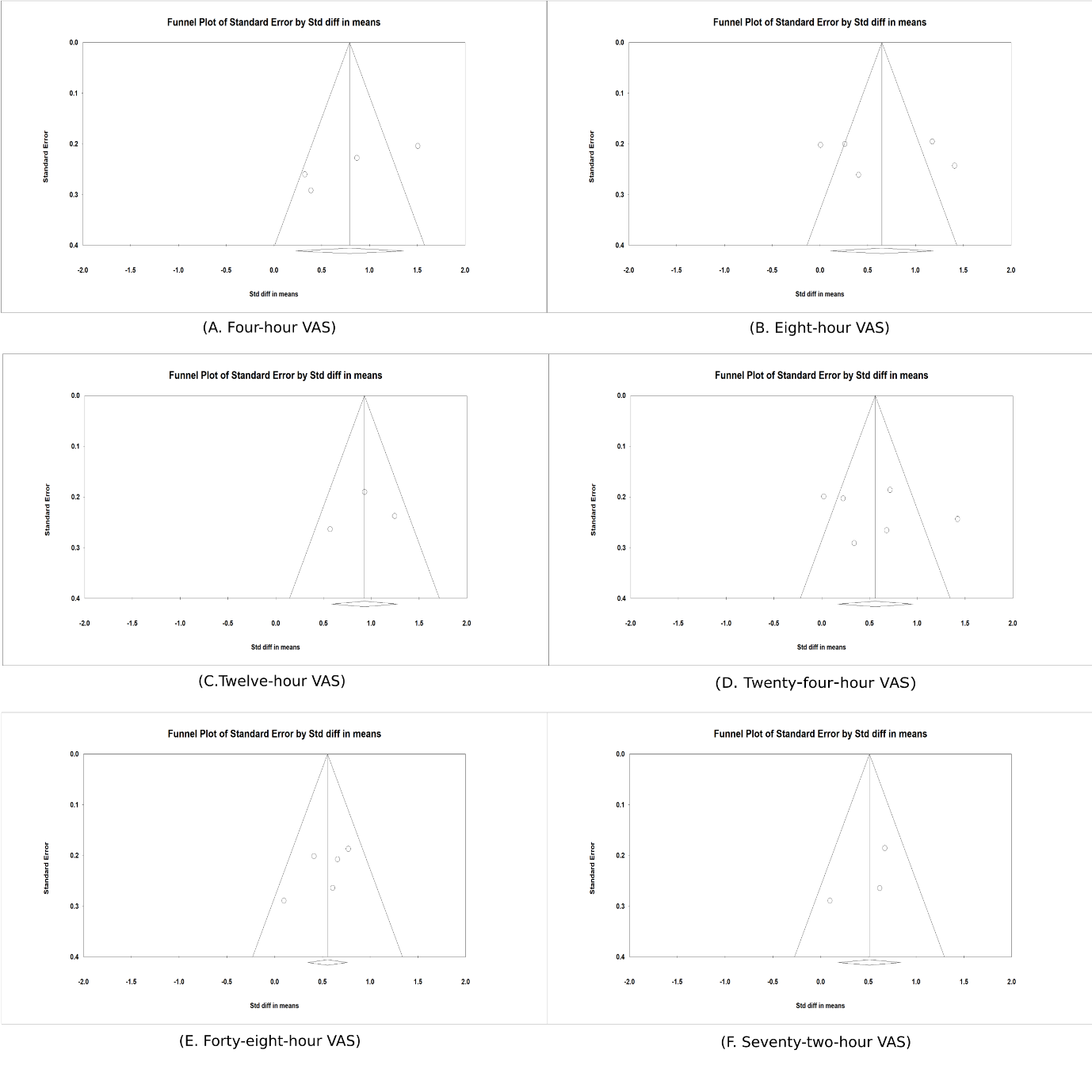

Supplement: Supplementary file 1 — Additional file 1. Quality of assessment for included studies using PEDro scale. [file 43019_2023_188_MOESM1_ESM.docx]
